# Correction: The role of AP-1 in self-sufficient proliferation and migration of cancer cells and its potential impact on an autocrine/paracrine loop

**DOI:** 10.18632/oncotarget.26636

**Published:** 2019-01-22

**Authors:** Sherif Abd El-Fattah Ibrahim, Aierken Abudu, Eugenia Johnson, Neelum Aftab, Susan Conrad, Michele Fluck

**Affiliations:** ^1^ Department of Microbiology and Molecular Genetics, Michigan State University, East Lansing, MI, USA; ^2^ Department of Histology and Cell Biology, Faculty of Medicine, Mansoura University, Mansoura, Egypt

**This article has been corrected:** The correct Author name is given below:

**Eugenia Johnson**

Original article: Oncotarget. 2018; 9:34259-34278. https://doi.org/10.18632/oncotarget.26047

