# Supplementary material for: The role of AP-1 in self-sufficient proliferation and migration of cancer cells and its potential impact on an autocrine/paracrine loop
Source: Oncotarget. 2018 Sep 28;9(76):34259–78. doi: 10.18632/oncotarget.26047 (PMC6188139; doi:10.18632/oncotarget.26047)
Supplement: Supplementary file 1 [file oncotarget-09-34259-s001.pdf]

The role of AP-1 in self-sufficient proliferation and migration of cancer cells and its potential impact on an autocrine/paracrine loop

SUPPLEMENTARY MATERIALS

Immunofluorescence

Approximately 0.5X10^5 MDA-MB-231/Flag-AFos cells were seeded in tissue culture chamber (Nalge Nunc International, Lab-Tek Chamber CVG. Cat. No.155380PK) on coverslips for 24 hours using DMEM with 10% FBS, then incubated with 0.05% FBS or 10% FBS DMEM for 48 hours followed by incubation with or without doxycycline (final concentration 50ng/ml) for 24 hours. Then the cells were fixed with 3.7% formaldehyde, permeabilized with 0.15% v/v Triton X-100 for 15 min and blocked in 2% w/v bovine serum albumin (BSA) in PBS for 1hr at room temperature. Cells were then incubated for 1h at room temperature with anti-Fra-1 antibody (sc-605x) (1:3000 dilution) in PBS containing 2% BSA. After that, cells were washed three times for 10 min each with PBS containing 2% BSA, followed by incubation with Alexa Fluor 488-conjugated anti-rabbit IgG (1:500 dilution) (Invitrogen) then washed three times for 10 min each with PBS. Next the cells were incubated with DAPI (final concentration 1μg/ml) (Sigma. Cat. No. D9542) for 10 min at RT and chambers mounted with Fluoro mount-G (Southern Biotech. Cat. No. 0100-01). Visualization and image analysis were acquired using an Olympus fluorescence microscope and Meta Morph software.

Preparation of nuclear and cytosolic extracts

Nuclear fractionation was performed as described previously [1]. Briefly, approximately 5X10^5 MDA-MB-231 cells were seeded in 6 well plate with DMEM containing 10%FBS for 24h. After that cells were washed twice with serum free medium, incubated with 0.05% or 10%FBS culture medium for 48h, then incubated with 10%FBS culture medium for various time periods from 1h to 24h. Cells were washed with PBS and then with ice-cold CSK buffer (10mM HEPES [pH 7.4], 300mM sucrose, 100mM NaCl, 3mM $MgCl_2$). Cells were scraped from plate, pelleted by centrifugation at 500 x g, and lysed in CSK-Triton buffer (CSK buffer containing 0.5% Triton X-100, protease inhibitor cocktail, and phosphatase inhibitors) at 10^{7}cells/ml for 10 min on ice. Nuclei were pelleted by centrifugation at 1,500 x g for 5 min at 4°C. Supernatants containing cytoplasmic and unbound nuclear protein, were removed and centrifuged at 16,000 x g for 10 min at 4°C to remove cell debris. The pelleted nuclei then were washed with 1ml of CSK-Triton buffer, pelleted by centrifugation at 1,500 xg for 5 min at 4°C, and re-suspended at 10^7 nuclei/ml in CSK-Triton buffer containing 160 U of DNase I/ml and 50mM MgCl2 and incubated on ice for 10 min. Nuclear remnants were then pelleted by centrifugation as before, and the proteins released into the supernatant. Then western blot was used to detect Fra-1 content at different components.

Co-immunoprecipitation (Co-IP)

Prepared nuclear and cytoplasmic lysates were used for Co-IP as described previously [2]. Briefly, complexes were immunoprecipitated with 1μg of normal rabbit IgG (sc-2027) or anti-Fra-1(sc-605x) polyclonal antibody for overnight at 4°C then 40μl of protein G agarose beads (Millipore. Cat. No. 16-266) were added for another 4 hours. The beads were washed three times with 1xPBS containing 0.1%Triton X-100. Proteins were eluted with SDS sample buffer and detected with western blot using anti-Fra-1 monoclonal antibody (sc-28310), anti-c-Jun monoclonal antibody (sc-74543) and anti-JunD monoclonal antibody(sc-271938).

RNA Isolation and Quantitative Reverse transcription-Polymerase Chain Reaction (RT-qPCR)

The RNA was extracted using the RNeasy kit (Qiagen). The complementary DNA (cDNA) generated was amplified using SYBR Green Real-time PCR Master Mix on an Applied Biosystems 7500 Real-time PCR System (Invitrogen). Expression levels were determined from the threshold cycle value using the method $2^{-\Delta\Delta Ct}$ with RPLPO expression as the reference control gene. Human RPLPO primers used were (forward) 5'-TGGTCATCCAGCAGGTGTTCGA-3' and (reverse) 5'-ACAGACACTGGCAACATTGCGG-3'. Human c-Fos primers used were (forward) 5'-AGAATCCGAAGGGAAAGGAA-3' and (reverse) 5'-CTTCTCCTTCAGCAGGTTGG-3'. Human Fra-1 primers used were (forward) 5'-AACCCTCCTCGC TTTGTGAG-3' and (reverse) 5'-GCTGGCTCTAC

TGTGAAGCA-3'. Human c-Jun primers used were (forward) 5'-TGGAAACGACCTTCTATGACGA-3' and (reverse) 5'-GTTGCTGGACTGGATTATCAGG-3'. Human JunD primers used were (forward) 5'-CGCCTGGAAGAGAAAGTGAA-3' and (reverse) 5'-GTTGACGTGGCTGAGGACTT-3'. The cycle conditions for the PCR were 1cycle of 5 minutes at 95°C and 40 cycles of 15 seconds at 95°C, 30 seconds at the annealing temperature 60°C, and 30 seconds at 60°C for extension [3].

REFERENCES

1. Burch PM, Yuan Z, Loonen A, Heintz NH. An extracellular signal-regulated kinase 1- and 2-dependent program of chromatin trafficking of c-Fos and Fra-1 is required for cyclin D1 expression during cell cycle reentry. Mol Cell Biol. 2004; 24:4696–709. https://doi.org/10.1128/MCB.24.11.4696-4709.2004.
2. Abudu A, Takaori-Kondo A, Izumi T, Shirakawa K, Kobayashi M, Sasada A, Fukunaga K, Uchiyama T. Murine retrovirus escapes from murine APOBEC3 via two distinct novel mechanisms. Curr Biol. 2006; 16:1565–70. https://doi.org/10.1016/j.cub.2006.06.055.
3. Chaturvedi LS, Basson MD. Glucagonlike peptide 2 analogue teduglutide: stimulation of proliferation but reduction of differentiation in human Caco-2 intestinal epithelial cells. JAMA Surg. 2013; 148:1037–42. https://doi.org/10.1001/jamasurg.2013.3731.

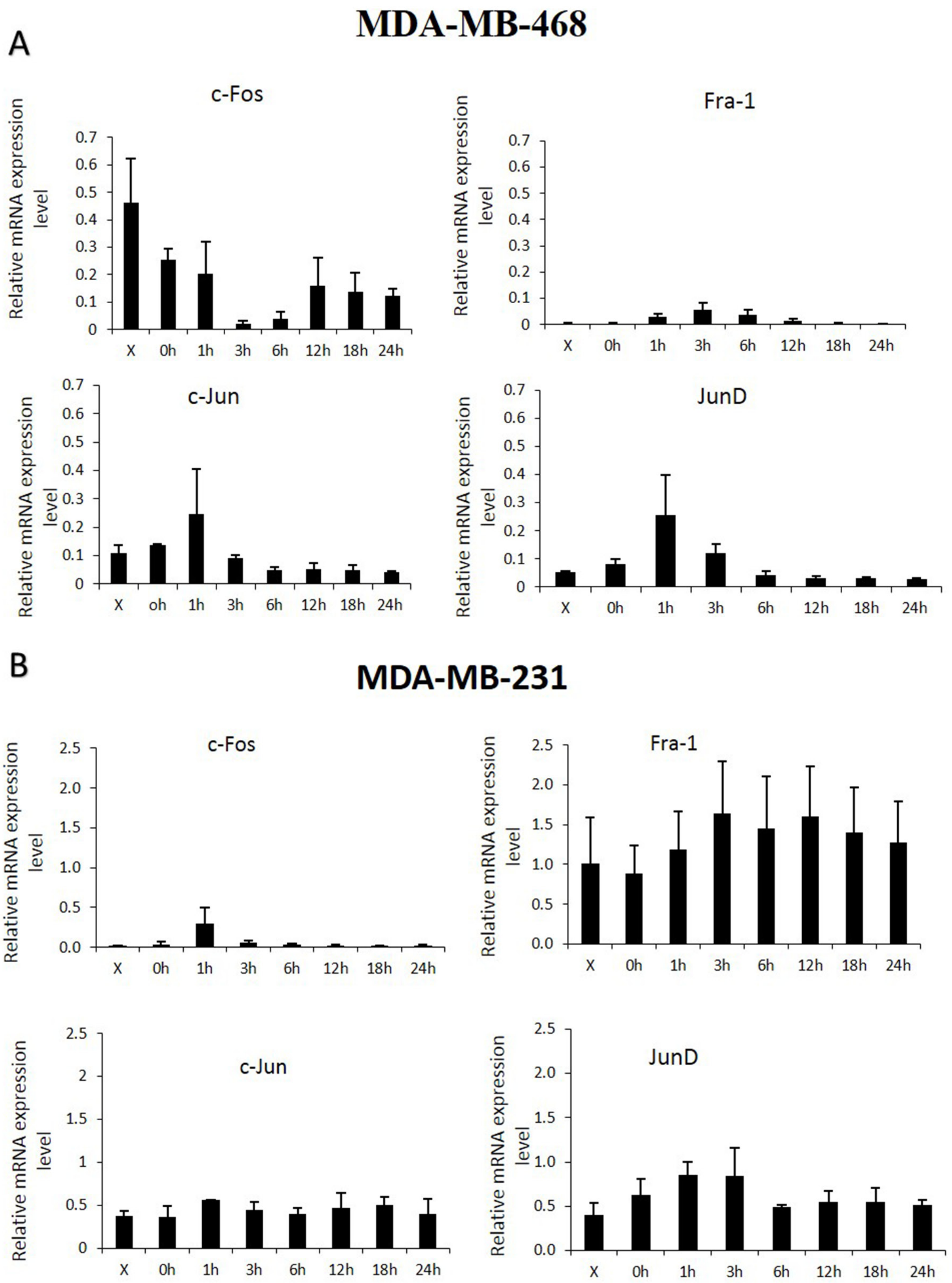

Supplementary Figure 1: Expression of AP-1 family members' mRNA in MDA-MB-468 and MDA-MB-231 cells in the presence or absence of serum. Serum starved cells were stimulated for the indicated time periods. The mRNA levels for Fra-1, c-Fos, c-Jun and JunD were analyzed using RT/qPCR in **(A)** MDA-MB-231 and **(B)** MDA-MB-468 cells. The mRNA levels were normalized to the house keeping gene RPLPO. The data shown is average of three independent experiments.

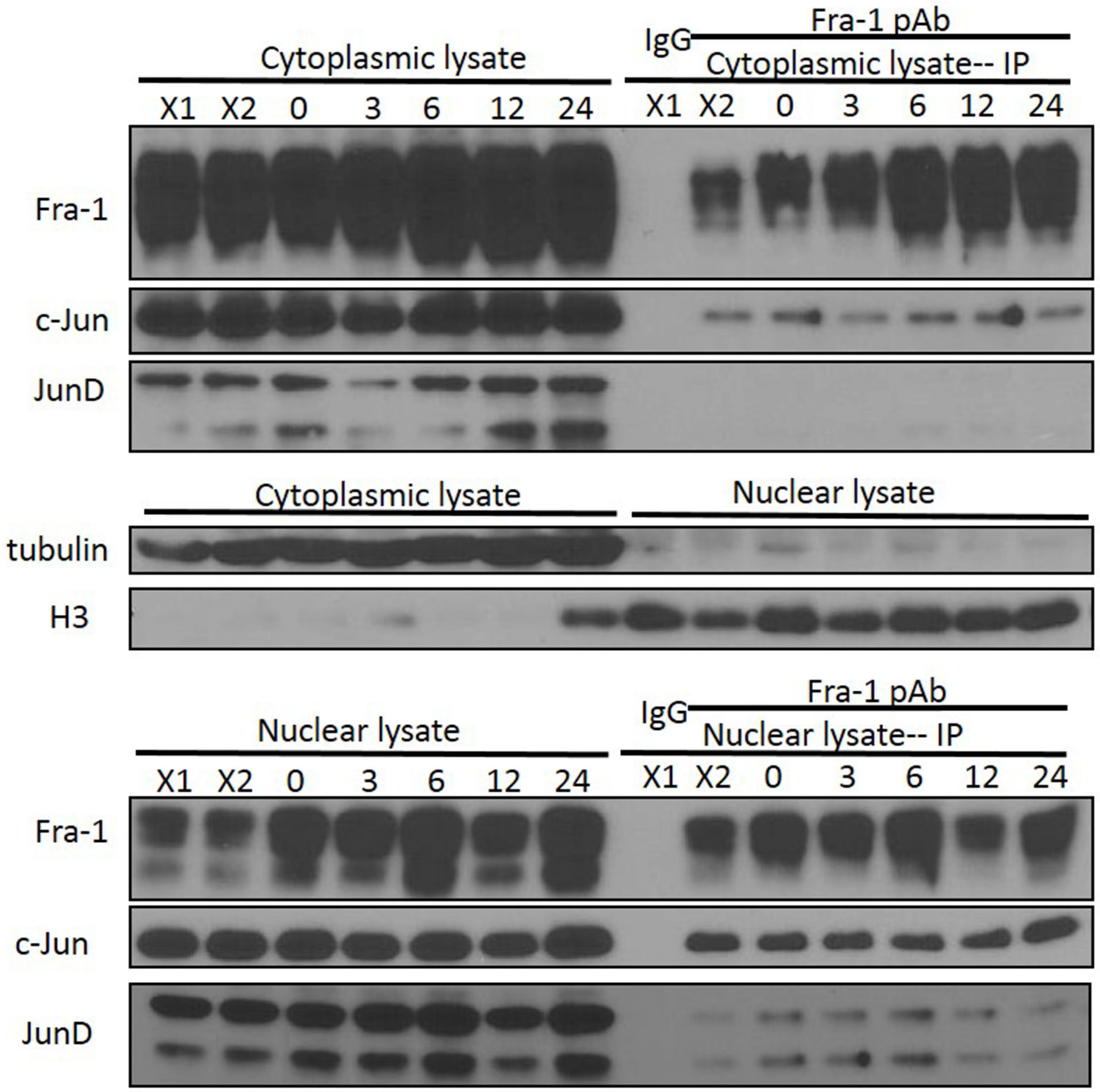

Supplementary Figure 2: Interaction of Fra-1 with c-Jun or JunD in presence or absence of serum in the cytoplasm or nucleus of MDA-MB-231 cells. Serum starved cells were stimulated with serum for the indicated time periods. Cytosolic and nuclear lysates were prepared and used for immunoprecipitation assays. Fra-1 protein was immunoprecipitated with anti-Fra-1 polyclonal antibody or non-specific IgG. Fra-1 immune complexes were analyzed by immunoblotting using anti-Fra-1 monoclonal antibody, anti-c-Jun monoclonal antibody and anti-JunD monoclonal antibody.

Cytoplasmic and nuclear extracts were analyzed by western blotting with these antibodies plus anti-α-Tubulin antibody, or anti-Histone-H3 antibody. The data shown are representative of three independent experiments.

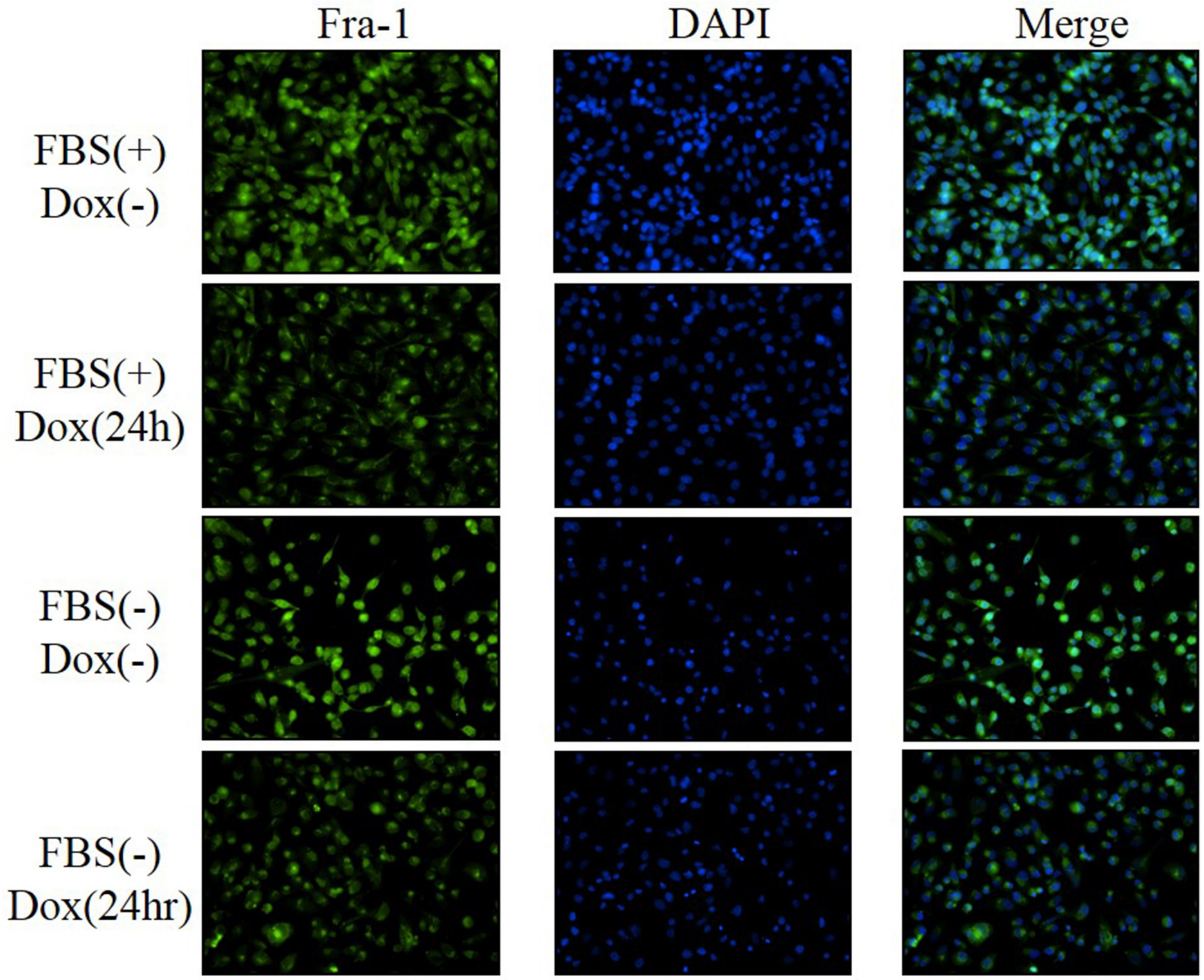

Supplementary Figure 3: Effect of induction of A-Fos on Fra-1 protein levels and localization in the presence or absence of serum in MDA-MB-231/Flag-AFos cells. Cells were immunostained for Fra-1 (left column, green) and nuclei were counterstained with DAPI (middle column, blue). The right column shows the merge image of the previous two columns. The top two rows show exponentially growing cells (FBS(+)) while those in the lower two rows correspond to serum starved cells (FBS(-)). A-Fos expression was induced for 24h (Dox (24)). White color indicates Fra-1 located in the nucleus. The data shown are representative of three independent experiments.
